# Tomato Pomace: Underestimated Sustainable Cosmetic/Pharmaceutical Raw Source

**DOI:** 10.3390/molecules31010053

**Published:** 2025-12-23

**Authors:** Ewa Maciejczyk, Anna Wajs-Bonikowska, Mirella Batory, Elzbieta Budzisz

**Affiliations:** 1 Institute of Natural Products and Cosmetics, Faculty of Biotechnology and Food Science, Lodz University of Technology, 90-530 Lodz, Poland; anna.wajs-bonikowska@p.lodz.pl; 2 Department of Cosmetology and Aesthetic Dermatology, Faculty of Pharmacy, Medical University of Lodz, 90-151 Lodz, Poland; mirella.batory@umed.lodz.pl; 3 Department of the Chemistry of Cosmetic Raw Materials, Faculty of Pharmacy, Medical University of Lodz, 90-151 Lodz, Poland; elzbieta.budzisz@umed.lodz.pl

**Keywords:** tomato pomace, molecular profile, sustainable raw material, dermal and pharmaceutical applications

## Abstract

This article explores the multifaceted potential of tomato pomace (TP) as a sustainable resource for the cosmetic and pharmaceutical industries, with a particular focus on the critical discussion surrounding peel–seed separation processes. Despite the significant volume of TP generated globally, valued molecules such as carotenoids, polyphenols, and high-quality oils remain underutilized. The separation of seeds from peels is highlighted as a critical step in the valorization of TP, as both components offer distinct physicochemical properties and bioactive constituents that significantly influence extraction efficiency and product quality. Various separation methods, including wet and dry techniques, have been innovatively developed; however, they present challenges such as resource consumption, operational complexity, and environmental concerns. The discussion advocates for a whole-pomace processing strategy that could streamline operations, enhance extraction efficiency, and create sustainable pathways for resource optimization. Additionally, the article highlights the importance of incorporating TP-derived compounds into cosmetic formulations and pharmaceutical products, which could lead to the development of new enzymes, antioxidants, and colorants that contribute to health and wellness. By championing the valorization of TP, the article advocates for a redefined perception of food waste, encouraging its utilization in sustainable practices that align with environmental goals.

## 1. Introduction

Tomatoes are recognized as one of the most widely consumed vegetables globally, as illustrated by the following numbers: around 180 million tons of tomatoes are cultivated worldwide each year, and more than 25% of this quantity is used for processing. Italy, Spain, and Portugal (respectively) are leading tomato producers in the European Union, with an annual harvest approaching 18 million tons (depending on the year) [1]. Typically, during the processing of these products, a by-product known as tomato pomace (TP) is generated. TP makes up 5% (*w*/*w*) of the fresh weight of tomatoes [2,3]. It comprises a mixture of pulp residue, peels, and seeds, which are undesirable in tomato products. The substantial volume of TP poses environmental challenges in terms of handling and disposal. However, this waste contains a range of beneficial compounds, including carotenoids, polyphenols, minerals, amino acids, proteins, and high-quality oil comprising valuable fatty acids and vitamins. This by-product is either discarded, utilized in agriculture, or added to animal food formulations [4,5].

Over the past decade, it has become evident that the food sector, encompassing food production and consumption activities, contributes significantly to global warming [6]. While efforts have focused on enhancing primary production (agriculture) and promoting dietary changes to mitigate the environmental impact, several studies have identified food waste reduction as an easily accessible measure to make a substantial difference [7,8,9,10,11,12,13]. The Food and Agriculture Organization (FAO) has highlighted that 1.3 billion tons of edible food are lost or wasted globally each year [14]. Recent research revealed that the EU-28 countries alone account for 88 million tons of edible and inedible food waste yearly [15]. The food sector must undergo significant changes, including improvements to supply chains and the implementation of effective food waste management, to achieve sustainability goals at both the European and global levels. Specifically, the United Nations’ Sustainable Development Goal (SDG) 12.3 aims to reduce food losses throughout production and supply chains by 2030 [16], while the EU-28 targets a 30% reduction in food waste by 2025 across the manufacturing, retail/distribution, food service, and household sectors [17]. These ambitious targets, along with the valuable content of food waste, have garnered attention from the industry and scientific community. By effectively capturing and repurposing unavoidable inedible food waste streams and residues, valuable biomolecules can be extracted to create new products, such as enzymes, antioxidants, proteins, nutraceuticals, cosmeceuticals, and colorants. This approach aligns with the goals of the European Bioeconomy Strategy, including ensuring food security, sustainable management of natural resources, reducing reliance on non-renewable resources, mitigating and adapting to climate change, and maintaining EU competitiveness. Recent studies on food waste biorefineries have revealed untapped potential in harnessing valuable biomolecules, leading to the production of high-value compounds [18].

Such food waste is tomato pomace, a by-product of the tomato industry. Although the problem is not new, as suggested by the 1917 report [2], it is only in recent decades that increased interest in the subject has emerged. The search phrase “tomato pomace” yields 470 results in Scopus (as of 31 January 2025), including 26 review articles, but only 9 are dedicated to tomato itself. The phrase “tomato by-product” was also examined and yielded 630 results, including 47 review articles, of which 19 were explicitly linked to tomatoes. In both searches, an upward trend in the number of documents was noted in the early 21st century. The identified trend aligns with the environmental concerns discussed in the preceding section. However, analyzing the above-mentioned review articles [19,20,21,22,23,24,25,26,27,28,29,30,31,32,33,34,35,36], it can be concluded that the discourse of using TP as a source of cosmetic raw materials is treated quite superficially. Nevertheless, the consideration of TP application in the pharmaceutical sector primarily centers on the use of lycopene. Thus, this article aims to provide a comprehensive review of TP, with particular emphasis on the technological and environmental implications of peel–seed separation, its chemical constituents, proven biological activities, and resulting applications in the cosmetic and pharmaceutical industries.

## 2. Why Is TP Produced?

To answer this question, it is best to refer to the structure of the tomato fruit itself. The tomato pericarp exhibits a radial arrangement in three layers: peel, red layer, and pericarp thickness [37]. The exocarp (peel) comprises a cuticle layer, a single layer of epidermal cells, and two to four layers of hypodermal cells with unevenly thickened walls [38]. In contrast, the mesocarp (red layer) consists of larger parenchymal cells compared to the exocarp. TP is the residue resulting from various tomato processing methods, predominantly composed of peels and seeds. The term “peel” refers to the outer layers of the tomato that are discharged during processing, primarily consisting of the exocarp and a small amount of concomitant mesocarp [39]. The composition of TP depends on the final product and peeling method. In canned tomatoes, TP comprises only peels without seeds, while in homogenized products like juice and paste, it includes discharged peels, seeds, and a small amount of pulp, representing 3–5% of the fresh fruit weight [2,3]. Peeling, a crucial operation, affects TP composition and final product yield, with seeds typically constituting 34–45% of TP on a dry basis [40,41,42].

## 3. Tomato Processing Methods

Efficient tomato peeling is a significant concern in the food industry and remains a key area of research for food scientists. Traditional industrial methods for tomato peeling involve hot lye and steam treatments. Hot lye peeling involves dissolving the outermost wax layer of tomatoes, allowing lye to penetrate the exocarp. This penetration results in the degradation of cell wall materials and the middle lamella between epidermal and hypodermal cells, facilitating the removal of tomato skins through friction or pressure washing [37]. Steam peeling, on the other hand, uses high-temperature steam to induce thermal-biochemical changes in the epidermis, affecting pectinaceous substances, polysaccharides, and cutin. Simultaneously, the heating effect generates internalized pressure in the tomato, separating the skin from the fruit body and rendering it easy to remove. However, due to environmental concerns about lye and the substantial energy consumption associated with steam, recent years have witnessed the exploration and application of environmentally friendly or energy-conserving tomato peeling methods. Notably, infrared-assisted peeling stands out for its water and chemical-free operation, yielding products with exceptional surface integrity and high firmness. Ohmic-assisted peeling, conducted in a sodium chloride solution, eliminates the need for lye. Ultrasonic-assisted peeling, by leveraging a hole effect, significantly reduces lye concentration. Due to its low-temperature nature, freeze–thaw-assisted peeling is beneficial for preserving the nutritive value of the final products. Although enzymatic-assisted peeling, which uses enzymes such as pectinases, cellulases, and hemicellulases, is economically and environmentally friendly, it faces high costs.

In addition to these innovative peeling methods, combining two or more of these approaches shows promise. For instance, ref. [43] indicates that infrared-assisted lye peeling surpasses both hot lye peeling and infrared-assisted peeling in terms of media volume and peeling loss, and yields results similar to ultrasonic-assisted hot lye peeling. From the perspectives of energy conservation, pollutant reduction, and profit improvement, there exists significant room for enhancement in the proposed tomato peeling methods. Future research in this area is warranted, given the potential environmental and economic benefits of refining and optimizing tomato peeling techniques and of reducing the ecological footprint of tomato processing in the food industry.

When discussing the tomato processing process, it is also necessary to mention modifications that enable the production of different product types. Two commercially employed techniques for the production of tomato preserves are “hot break” and “cold break”. The “break” phase holds significant importance in tomato processing, to the extent that it is regarded as a crucial factor in selecting the tomato variety for production. During this stage, the tomatoes undergo rapid heating. The term “hot” typically denotes a chopping temperature ranging from 85 to 90 °C, which leads to the deactivation of enzymes crucial for aroma and viscosity, such as pectin methylesterase and polygalacturonase, which break down pectin chains in tomato tissue. By inactivating these enzymes during the hot break process, a more viscous product can be obtained, as desired. On the other hand, lipoxygenase, the enzyme responsible for the formation of key fresh aroma compounds by breaking down unsaturated fatty acids, is inactivated during the hot break, leading to fewer aroma compounds. A “Cold” break, conversely, refers to a temperature below 70 °C, which promotes enzyme activity. A distinctive feature of the cold break process is a reduction in viscosity. Cold breaking offers advantages over hot breaking, with the final product reportedly exhibiting a more natural color and a fresher flavor. The hot break method remains the predominant method for producing most tomato products, which helps maintain high viscosity. Nevertheless, an increased adoption of the cold break method is expected to yield tomato products with a more vibrant aroma [44,45,46].

In numerous investigations on TP composition [3,4,42,47,48,49,50,51,52,53,54,55,56,57,58,59,60,61,62,63,64,65,66,67,68,69,70,71,72,73,74,75,76,77,78,79], only a minority of researchers have specified the processing methodology (hot or cold break) used to obtain the pomace used in their studies [49,66,67,68,69]. Furthermore, it is imperative to acknowledge the ongoing advancements in tomato processing technology over the years.

## 4. Molecular Composition of TP Fractions (Peel vs. Seed vs. Whole Pomace)

TP constitutes a reservoir of valuable components, prominently including lycopene, dietary fiber, protein, and oil. The distinct components present in the seed and peel segments underscore their diverse nutritional profiles. As mentioned in Section 3, numerous publications have addressed the components of TP; therefore, this section provides a summary of existing knowledge on this topic. However, to avoid referring readers to other publications, summary tables and a brief commentary are provided here.

Tomato peel is notably abundant in dietary fiber, lycopene, and phenols, whereas the seed primarily comprises oil and protein. In particular, tomato peels exhibit a notable potassium content, averaging approximately 1.1 g per 100 g, with sodium levels relatively lower at 70 mg per 100 g. This characteristic contributes to a low Na/K ratio, rendering tomato peel a potentially beneficial agent in combating cardiovascular diseases [60].

Whole TP is also abundant in minerals, particularly calcium (Ca), phosphorus (P), magnesium (Mg), sodium (Na), and potassium (K). In contrast, the content of iron (Fe) and zinc (Zn) was relatively low [54,55,56,60,62,79].

- Lycopene

**Figure 1:**
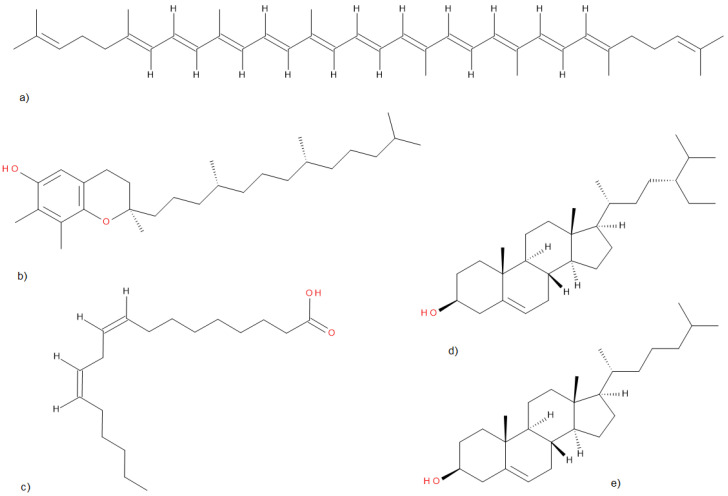
Tomato pomace’s main lipophilic components are (**a**) lycopene, (**b**) tocopherol, (**c**) linoleic acid, (**d**) β-sitosterol, and (**e**) cholesterol.

The proximate compositions of the entire TP and its components, namely peels and seeds, are presented in Table 1, and the structures of selected compounds are shown in Figure 1. As expected, tomato peels are the richest reservoir of lycopene, comprising 80–90% of the overall carotenoids, as reported by Nour et al. [62]. For comparison, the lycopene content in 100 g of fresh tomato is 4.96–6.67 mg [80,81,82], and in pomace—0.48–8.03 mg/100 g (wet weight) [63], which suggests, depending on the pomace batch, that the vast majority of lycopene ends up in production waste: pomace. Lycopene possesses significant antioxidant properties, offering robust protection against UV rays when applied topically. Due to these attributes, this compound plays a crucial role among substances intended for topical application [83,84].

- Oil and fatty acids

Tomato seeds, followed by tomato pomace, were particularly rich in oil (16–24%, 2–23%, respectively), whereas tomato peels showed a low oil content (1.5–4.9%). Regardless of the oil source, namely pomace, seeds, or tomato peel, the predominant fatty acid identified was linoleic acid (C18:2), comprising concentration ranges of 51–53, 37–57, and 42–52% in pomace, seed, and peel oil, respectively (Table 2). Szabo et al. [85] reported an extremely high linoleic acid content (up to 92.9%). However, the TP used by them was obtained in the laboratory from fresh tomatoes. Palmitic acid (C16:0) emerged as the predominant saturated fatty acid in many researchers’ studies, ranging from 2% to 25%. Based on these findings, tomato oils belong to the linoleic-oleic acid oils category and might be used as an edible oil with high nutritional quality.

**Table 1:** Proximate compositions of the entire TP, peels, and seeds.

Material	Oil [%]	Lycopene [mg/100 g dw]	Proteins [% dw]	Total Phenolic Content [mg GAE/100 g dw]	Fibers [% dw]	Ash [% dw]	Reference
Pomace	8.83		20.14	5510	64.12	7.01	[56]
	5.85		19.27		59.03	3.92	[53]
	2.10		16.10	14.6	46.2	4.1	[86]
	21.80						[78]
	2.19	51.06	17.62	122.95	52.44	4.21	[62]
	8.52		14.95	179.67	39.45	4.27	[79]
	9.87 ^a^		24.67			5.29	[87]
	8.37–16.24	9.816–17.21 ^b^	15.08–22.70		48.49–64.75 IDF 8.91–10.04 SDF	2.88–4.40	[88]
	11.17–16.73	27.99–69.09	16.81–23.25	10.08–22.75	48.62–53.97	3.33–4.02	[41]
	19–23	14.9–28.8		111.9–407.7			[85]
Peel	1.77	3.67	14.47	4683	48.52	5.74	[56]
				5312			[50]
		193.00	<3%	51.40 ^c^			[65]
	4.04		10.50			5.90	[60]
	1.5–1.98		0.99–1.70		57.7–66.3	1.9–3.0	[61]
	4.53–4.90		9.82–10.21		54.27–62.63 IDF 5.72–14.0 SDF	4.51–5.76	[89]
Seed	17.15		25.50	2700	54.24	4.61	[56]
	13.30–19.30						[58]
	22.10		32.10	10.00–12.30	16.10	5.10	[86]
	22.40		32.60		14.80	4.80	[57]
	24.57		23.60			3.64	[64]
	16.33–23.37						[67]
	17.83		27.24			3.37	[59]
	17.90		38.40		17.00	1.9	[3]

Description: ^a^—wet weight; ^b^—not mentioned dry or wet; ^c^—mg GAE/L; IDF—insoluble dietary fiber; SDF—soluble dietary fiber.

**Table 2:** The relative concentration (%) of the FA from tomato seed oil.

Material	C10:0	C12:0	C14:0	C16:0	C16:1	C17:0	C17:1	C18:0	C18:1	C18:2	C18:3	C18:4	C20:0	C20:1	C22:0	C24:0	Reference
Pomace	nd	nd	0.2	14.00	0.50	0.30	nd	6.00	22.60	53.60	2.00	nd	0.30	0.10	tr	0.10	[78]
	nd	nd	0.41	16.32	0.64	nd	0.52	5.43	18.50	51.91	3.35	0.48	nd	nd	nd	0.29	[62]
Seeds	nd	nd	0.12–0.31	13.92–24.82	0.05–0.84	0.07–0.14	nd	0.46–1.07	8.60–20.89	47.85–72.69	2.28–6.34	nd	0.27–0.55	nd	nd	nd	[58]
	nd	nd	0.50	13.80	0.50	0.60	nd	3.40	21.40	55.00	4.00	nd	0.50	nd	nd	nd	[57]
Peel	nd	nd	0.34	15.19	1.82		nd	6.84	19.14	52.41	4.26	nd	nd	nd	nd	nd	[60]
	1.30	1.00	3.00	20.60	1.80	3.10	nd	4.70	16.30	42.10	2.70	nd	2.50	nd	nd	nd	[57]

nd—no data.

Interestingly, tomato seed oil obtained from the cold break process exhibited higher oleic and linoleic acid contents compared to the hot break seed oil. Conversely, the linoleic acid content was lower in the cold break seed oil than in the hot break seed oil. Additionally, the hot break seed oil contained a higher total saturated fatty acid content, whereas the cold break seed oil had a higher total unsaturated fatty acid content [50]. These findings were not observed in further investigation [70].

- Sterol fraction

Total sterol content in tomato seed oil varied between 1.55 and 12.30 mg/g [3,69,78,90]. The reported sterolic fraction of the tomato seed oil was composed mainly of β-sitosterol (predominant), campesterol, cholesterol, stigmasterol, and Δ^5^-avenasterol or cycloartenol, with trace amounts of several other phytosterols (Table 3) [69,78,90]. The elevated cholesterol content, compared to other vegetable oils, which typically contain minimal amounts of this sterol, is a distinguishing feature of phytosterols found in seeds from the Solanaceae family [91,92].

**Table 3:** Composition of sterol fraction of tomato seed oil.

Compound	%				
Cholesterol	5.2–5.6	9.0	15.00	9.62	7.5
Cholestanol	0.7–1.0	tr	nd	nd	nd
Lathosterol	1.0	nd	nd	nd	nd
Brassicasterol	0.3–0.5	nd	1.50	nd	nd
24-Methylenecholesterol	nd	1.0	1.20	nd	0.3
Campesterol	4.8–5.2	5.0	6.70	2.67	5.7
Dihydrolanosterol	3.9–4.5	nd	nd	nd	nd
Stigmasterol	10.4–11.0	9.0	14.40	6.54	11.3
Δ^7^-Campesterol	nd	nd	0.30	nd	0.8
Clerosterol	nd	nd	tr	nd	0.9
β-Sitosterol	33.0	63.0	52.00	31.08	58.4
Cycloartanol	22.0	55.0	nd	nd	nd
Δ^5^-Avenasterol	nd	nd	6.70	nd	9.5
Δ^7,24^-Stigmastadienol	nd	nd	0.50	nd	0.9
Δ^7^-Stigmastenol	nd	nd	0.40	nd	0.2
Δ^7^-Avenasterol	nd	nd	0.10	nd	0.2
Erythrodiol	nd	nd	0.10	nd	nd
β-Amyrin	1.0–1.4	tr	nd	nd	nd
Citrostadienol	1.7	nd	nd	nd	nd
Reference	[90]	[92]	[78]	[59]	[3]

nd—no data; tr—trace amount; digits do not require explanation.

- Tocopherols

Eller et al. [90] reported that the total tocopherol content varied from 0.94 to 1.11 mg/g in tomato seeds, depending on the extraction method. These values fall within the same range as most crude vegetable oils such as soybean and sunflower oil [78]. γ-Tocopherol was the predominant homolog, with significantly lower amounts of α- and δ-tocopherol [3,78,90]; however, Lazos et al. [78] noted a notably higher quantity of δ-tocopherol. The authors suggested that the higher oxidative stability of tomato seed oil compared to sunflower oil can be attributed to components in the non-glyceride fraction of the oil that exhibit antioxidant properties [78].

The proximate composition of tomato byproducts showed that seeds have the highest amount of protein, followed by pomace and peels. The ash and crude fiber content were at similar levels in tomato pomace, peels, and seeds.

- Phenolic fraction

The reported total phenolic content was the highest in TP (up to 408 mg GAE/g), followed by peel (up to 53.12 mg GAE/g) and seeds (up to 27.00 mg GAE/g). These values were strongly dependent on tomato variety, as shown by Szabo et al. [85]. However, in this study, pomace was prepared in the laboratory. The research evaluated the carotenoid, phenolic, and amino acid contents of ten tomato cultivars’ processing wastes and their correlation with antimicrobial and antioxidant properties. The extracts exhibited notable antibacterial activity against Gram-positive bacteria, such as *Staphylococcus aureus*, with this activity closely linked to the amount of isochlorogenic acid in each tomato cultivar. Additionally, all genotypes demonstrated high antioxidant activity, with the Tiny Tim cultivar showing significantly higher levels of flavonol glycosides and isochlorogenic acid compared to other varieties.

- Factors influencing the composition

Moving toward the summary of this section, Chandra et al. [93] revealed that the antioxidant potency of different parts of tomato fruit followed the order of Skin > Pulp > Seeds. Overall, the study demonstrated that high-altitude cultivars possessed superior antioxidant capacities. However, these findings were based on studies conducted using fresh tomato material.

Another determinant of tomato pomace composition is tomato ripeness, which can be divided into two stages: red-ripe and breaker-ripening. The study by Georgaki et al. [81] reveals variations in the content of fat, protein, lycopene, and phenolic compounds among different tomato varieties at various ripening stages.

Consequently, these studies indicate that genetic factors, the production site, ripeness, and extraction method significantly influence the antioxidant levels and activity of tomato genotypes. Therefore, selecting the appropriate genotype is crucial for maximizing health benefits. Nevertheless, from the perspective of the processing industry, this poses a potential challenge. Empirical evidence suggests that companies producing tomato-based products often overlook the diversity of tomato cultivars. It is plausible that future dissemination of research findings could foster greater awareness within the processing industry, facilitating the selection and procurement of cultivars characterized by elevated levels of health-promoting constituents at the proper ripening stage.

- Current state and future perspective

Tomato pomace provides ingredient-grade fractions with distinct molecular profiles (peel vs. seed vs. whole pomace), and the choice of separation/extraction path predictably shapes the stability and composition of quality control (QC) markers (total lycopene + %cis; PV/AV/TOTOX; sterols/tocopherols; FA/TAG). TP bioactive compounds extraction routes and cosmetic/pharmaceutical applications are summarized in Table 4. Additionally, consumers are making more and more conscious choices and have increased their interest in natural ingredients [94]. In future perspectives, priorities include (i) standardization of quality control panels and acceptance intervals, (ii) scaling of green extractions (EtOH/H_2_O, SCO_2_) with isomerization/oxidation control, (iii) structure–property–function mapping for dermal/pharmaceutical applications, and (iv) harmonization of INCI labeling for fractions derived directly from TP.

**Table 4:** Bioactive compounds from tomato pomace: extraction routes and cosmetic/pharmaceutical applications.

Compound/Class	Source Fraction (TP)	Extraction Route(s)	Stability/QC Notes	Cosmetic/Pharmaceutical Applications (Examples)	Evidence Level (In Vitro/In Vivo/Clinical/Regulatory)	Key Refs.
Lycopene (carotenoid)	Peel; Whole pomace	EtOH/H_2_O; Supercritical CO_2_ (±modifiers); maceration; enzyme-assisted wet split → extraction; ultrasound- and microwave assisted extraction	Heat/oxygen sensitive; trans→cis isomerization	Antioxidant/colorant; dermal antioxidation adjunct; potential photoprotection support	in vitro, in vivo; regulatory discussion in the literature	[30,41,56,62,63,65,83,84,85,88]
(β-Carotene (carotenoid)	Peel	EtOH/H_2_O; SCO_2_; maceration	Degrades with heat/O_2_; report with HPLC-DAD	Antioxidant/colorant; supportive dermal formulations	in vitro; limited in vivo	[62,95,96,97,98]
Naringenin (phenolic)	Peel; Seed	EtOH/H_2_O; solvent-optimized extraction; ultrasound- and microwave assisted extraction	pH/solvent dependent; co-extracts with other phenolics	Antioxidant/anti-inflammatory potential (model systems)	in vitro	[54,70]
Rutin (phenolic)	Peel; Seed; Whole pomace	EtOH/H_2_O; ultrasound- and microwave assisted extraction	Sensitive to hydrolysis; monitor with UPLC-MS/HPLC-UV	Antioxidant; capillary-strengthening claims (literature, non-TP)	in vitro; limited in vivo	[54,62,70]
Seed oil (TAG/FA matrix)	Seed oil	Cold pressing; Soxhlet (n-hexane/EtOH); SCO_2_; pressurized liquid extraction	Track PV/AV/TOTOX; α/γ-tocopherol as endogenous antioxidants	Emollient; lipid vehicle; dermal barrier support	in vitro; GRAS for edible seed oils (general)	[57,58,60,62,78]
α-/γ- Tocopherol (vitamin E)	Seed oil	Co-extracted with oil; HPLC-FLD profiling	Depletes with oxidation	Antioxidant; skincare formulations	in vitro; widespread cosmetic use (non-TP specific)	[3,78,90]
Phytosterols (β-sitosterol, campesterol, stigmasterol)	Seed oil	Saponification → GC-MS profiling; co-extracted in oil	Relatively stable; losses during refining	Skin barrier support; anti-inflammatory potential in the literature	in vitro; some in vivo (non-TP)	[3,70,79,88]
Squalene	Pomace	Saponification → GC-MS profiling; co-extracted in oil		Skin barrier support	in vitro; some in vivo (non-TP)	[96]

## 5. Seeds and Peels: To Separate or Not?

TP is composed of a complex matrix of seeds and peels, each with distinct physicochemical properties and bioactive constituents. As some researchers suggest, separation of these components is a critical step in the valorization of TP, as seeds and peels exhibit differential compositions that influence extraction efficiency and final product quality [19,31,56,88,99,100,101]. Various separation techniques have been developed and are broadly categorized into wet and dry methods [39,88,101]. This part critically evaluates these techniques, highlighting their advantages, limitations, and industrial applicability.

Wet separation exploits the density differences between tomato seeds and peels. The process involves mixing TP with water in a mixer-settler system, where denser seeds sink to the bottom while the peels remain buoyant. Kaur et al. [39] developed a flotation system that achieved separation efficiencies of 69.17% and 48.29% for peels and seeds, respectively. Similarly, Shao et al. [88] refined a laboratory-scale wet separation technique, demonstrating that iterative processing enhanced separation purity to 89.65% for peels and 96.6% for seeds. However, the wet separation method presents several challenges, including significant micronutrient losses due to leaching, high water consumption, and the generation of wastewater, which poses environmental concerns.

Dry separation methods involve an initial drying step, followed by mechanical fractionation using air classifiers or cyclones. In this approach, dried TP is introduced into a cyclone, where aerodynamic forces separate the components based on their weight. Peels, being lighter, are carried upwards and exit through the upper outlet, while denser seeds descend against the airflow and exit through the lower outlet. Shao et al. [101] optimized this process using an air aspirator system and response surface methodology (RSM), achieving a separation efficiency of 68.56% at an air velocity of 6.4 m/s and a feed rate of 40 kg/h. Despite its advantages, dry separation is associated with drawbacks, including the generation of dust, which can be hazardous to workers and contribute to air pollution. Additionally, the formation of agglomerates, where seeds adhere to peels, complicates complete separation.

Although the separation of seeds from peels is widely advocated in laboratory studies, its scalability remains a challenge. Many methodologies, such as those employed by Shao et al. [88,101], rely on laboratory-specific modifications (e.g., specialized mills with taped blades) that are not directly translatable to industrial-scale operations. The need for dedicated equipment increases operational costs, making large-scale implementation less viable.

In the broader context of TP utilization, researchers and industry stakeholders seek efficient strategies for lycopene extraction. Numerous patents [102,103] outline various pretreatments, solvent systems [104], and unconventional extraction processes [105,106,107,108] aimed at maximizing carotenoid recovery. However, these methods are often multi-step and resource-intensive, limiting their practical application. Isolation methods of pure lycopene from pomace definitely requires further optimization in the context of applications, e.g., pharmaceuticals. Notably, some sources claim that 90% of carotenoids used in industry are still chemically synthesized [109], indicating a gap between research findings and industrial implementation. However, more recent reports suggest that the figure is around 60% [110].

From an economic and environmental standpoint, TP processing should be cost-effective and energy-efficient. Given that TP naturally contains valuable bioactive compounds, process optimization should minimize resource inputs while maximizing yield. Studies indicate that supercritical lycopene extraction is enhanced when vegetable oil is used as a solvent, and TP seeds serve as a natural source of oil. Additionally, in industrial settings, TP exists in processing lines at elevated temperatures (60–80 °C), suggesting that in-house drying and direct extraction could be a feasible approach. Additionally, research shows that the supercritical extraction of lycopene is more effective when using a vegetable oil co-solvent [52]. Vegetable oil co-solvents not only enhance extraction but also help protect lycopene from degradation during both extraction and storage, resulting in a longer shelf life and higher-quality extracts [111].

Considering these factors, it may be beneficial to reconsider the widely endorsed practice of separating seeds from peels before extraction. Instead, a whole-pomace processing strategy could streamline operations, enhance extraction efficiency, and reduce processing costs. Both peels and seeds contain valuable bioactives, and integrating these components within a biorefinery framework aligns with sustainable production goals. Almeida et al. [13,26] provide an extensive discussion on the biorefinery approach to valorizing tomato waste, advocating integrated processing strategies that enhance resource efficiency.

To conclude, the separation of tomato seeds and peels is a well-researched topic, yet industrial-scale implementation remains challenging. While wet and dry separation methods offer distinct advantages, they also present significant limitations, particularly in terms of resource consumption and operational complexity. Given the inherent value of both TP fractions, an alternative whole-pomace processing approach may offer a more sustainable and economically viable solution. Future research should focus on optimizing this strategy within an industrial context, integrating it into broader biorefinery frameworks to maximize TP valorization while minimizing environmental impact (water/energy/solvents).

## 6. New-Old Problem: Extraction By-Product

Defatted TP, obtained after extracting the lipophilic fraction, represents a nutrient-rich secondary raw material characterized by a high protein content (often exceeding 20% dry matter) and substantial levels of dietary fiber (frequently surpassing 50% dry matter). Additionally, it contains significant concentrations of essential minerals, particularly potassium (K), phosphorus (P), calcium (Ca), and magnesium (Mg), as well as a diverse array of bioactive compounds. Over the past two decades, scientific investigations and industrial reports have consistently highlighted the considerable potential of this by-product as a functional feed and food additive, as well as a promising source of nutraceuticals [32,36,112,113].

The removal of the lipid fraction enhances the oxidative stability of the remaining material, thereby improving its applicability in various industrial processes. Specifically, defatted TP can serve as a substrate for the production of protein-rich feed concentrates, fiber-based food additives, or the extraction of polyphenols with antioxidant properties [32,102,114,115,116]. Extensive literature reviews confirm that TP constitutes a valuable secondary resource abundant in nutrients and bioactive compounds with health-promoting effects. Moreover, the defatted residue has potential applications in the cosmetic industry, particularly as an exfoliating agent.

Beyond its applications in food, feed, and cosmetics, defatted TP also holds promise as an agro-industrial input and an alternative energy source. In agricultural contexts, it can be utilized as an organic soil amendment, enhancing soil structure, fertility, and nutrient availability for crops [117,118,119,120,121]. Furthermore, its high organic matter content makes it a viable candidate for renewable energy production, whether through anaerobic digestion for biogas generation, combustion in pelletized form for heating applications, or as a feedstock for liquid biofuel production [91,122].

Utilizing TP in these diverse applications not only mitigates waste disposal challenges but also contributes to environmental sustainability by reducing ecological burdens while generating energy and closing material loops, aligning with circular economy principles. Empirical evidence from both scientific research and industrial practice underscores the feasibility and economic viability of this integrated approach, demonstrating concurrent environmental and economic benefits.

## 7. The Use of Tomato Products in the Pharmaceutical Industry

Humans cannot synthesize lycopene on their own; therefore, it must be obtained through food or supplementation. In the pharmaceutical industry, lycopene obtained from extracts from the fruit of the tomato *Lycopersicon esculentum* L. (*Solanum lycopersicum* L.) is used, although the tomato species is not always specified. Lycopene is not available as a medicine, but there are available dietary supplements containing lycopene—over 137 in various forms (capsules, tablets, chewable tablets, drinking liquids, powders, jelly tablets, oils). This compound is often found in preparations labeled as E160d.

- Lycopene supplements

The register of products reported to the Chief Sanitary Inspector (Register of products covered by notification of the first introduction to the market—https://e.sanepid.gov.pl/spoz/rpop, URL accessed on 26 April 2024) shows that there are 137 dietary supplements with lycopene on the Polish market (5087 products in the world) and 35 supplements containing tomato as an ingredient—in Poland (11,187 products in the world). Due to the fact that the register does not specify the amount of lycopene in preparations, the pharmaceutical market was reviewed in this respect.

Supplements with lycopene (Table 5), according to manufacturers’ declarations, have strengthening, cleansing and protective effects. They are used to control inflammatory responses and the proper functioning of the immune system. They also have an antioxidant effect. They are used to prevent many cardiovascular diseases, including atherosclerosis and hypertension, for the proper functioning of the eye, and for the healthy functioning of the prostate. Lycopene is also included in vitamin and mineral complexes for men and women. It is also available as a separate preparation—lycopene.

- Bioavailability, Antioxidant Mechanisms, and Dose-Dependent Redox Behavior

In order to check the declarations of dietary supplement manufacturers, evidence was found confirming their effectiveness. The mechanism of lycopene absorption is not fully known. Studies have shown that lycopene from tomato products enters the bloodstream more easily if the tomato is heated and if a source of fat is included in the meal. According to a study, plasma lycopene concentrations increased only slightly in the group receiving 180 g of tomato juice (containing 12 mg of lycopene) daily for 6 weeks [123]. Other studies have proven that lycopene is characterized by the highest antioxidant activity in the group of carotenoids, significantly exceeding β-carotene, tocopherol, β-cryptoxanthin, lutein, and zeaxanthin [124,125]. Lycopene is also the strongest scavenger of free radicals and singlet oxygen due to its long chain with conjugated double bonds [124,126,127,128]. To produce reactive oxygen species (ROS), lycopene works in three ways: first it is radical addition, i.e., the formation of an adduct, then the transfer of an electron to a radical, and finally the abstraction of allyl hydrogen [124]. In small doses, lycopene acts as an antioxidant, while in large doses it acts as a pro-oxidant. If lycopene acts as a pro-oxidant in previously damaged cells, it may help prevent the formation and progression of cancer lesions, as well as cancer cytotoxicity [124,129,130].

- Lycopene in Humans

A diet rich in lycopene may also help prevent or reduce the risk of cardiovascular disease. According to research, for preventive purposes, it is enough to use 5 to 7 mg of lycopene per day, while higher doses of 35–75 mg/day can be administered in the event of cardiovascular diseases or cancer [124,131,132,133]. When combined with prostaglandins and phospholipids in cell membranes, lycopene can improve the skin’s defense mechanisms. Lycopene has also been used to conduct research related to the prevention and treatment of other ailments, such as inflammatory diseases, cancer, vitamin A deficiency, cardiovascular diseases, bacterial infection, skin diseases (Photodamage by UV-B, Atopic dermatitis, Photo aging) [124].

The conducted research also showed that the risk of developing prostate cancer was halved in the group of men with high serum lycopene concentration (approximately 33 mg/day) compared to the group with lower concentration (approximately 13 mg/day). A high intake of lycopene is associated with a lower angiogenesis potential, which reduces the rate of cancer progression, especially of higher malignancy, and reduces mortality. The results of clinical trials suggest that lycopene supplementation at a dose of 15–30 mg/day reduces the incidence of benign prostatic hyperplasia and prostate cancer [134,135].

However, studies of premenopausal women show that the risk of breast cancer is associated with high IGF-1 concentration, and the amount of consumed lycopene reduces the concentration of IGF-1 circulating in the blood by stimulating the synthesis of the IGF-1 binding protein [136,137]. High serum lycopene levels are also associated with a lower risk of ovarian and endometrial cancer. The study results suggest that women with high serum lycopene concentration (0.59–1.58: g/dL) had an 85% reduced risk of endometrial cancer compared to patients whose serum lycopene concentration was 0.36–0.51 g/dL [138]. Additionally, it has been shown that lycopene used during radiotherapy in women with breast cancer has a protective effect and reduces the side effects of radiation on the irradiated skin [139].

High blood pressure, cholesterol, and smoking are the main risk factors for cardiovascular disease. Damage and reconstruction of blood vessels impede blood flow, and atherosclerosis is the most common cause of cardiovascular diseases that attack the heart and brain [120,136]. A direct relationship has been demonstrated between high levels of LDL lipoproteins and cholesterol and the formation of atherosclerotic plaque. Studies conducted on large groups of patients with diagnosed cardiovascular diseases showed that the level of lycopene is significantly lower than in the control group [137]. VCAM-1 (Vascular cell adhesion protein-1) and LDL were found to be inversely associated with serum lycopene. Lycopene supplementation can improve microcirculatory function by reducing the concentration of sVCAM (Circulating Vascular Cell Adhesion Molecule) and sICAM (Soluble intracellular adhesion molecules), reducing DNA damage, and increasing superoxide dismutase (SOD) activity [120,138,139,140]. This level is also lower in smokers than in non-smokers. It is suggested that this situation occurs due to the increased consumption of antioxidants in smokers due to increased oxidative stress. It is believed that lycopene circulating in the plasma protects against atherosclerosis, especially in smokers [120,141,142]. Lycopene supplementation has been shown to increase blood lycopene levels, reduce oxidative stress markers, and improve antioxidant status (TAS) [120]. According to studies, lycopene intake and the thickness of the intima-media of the carotid artery, which is a risk factor for cardiovascular diseases, are inversely related [120,143]. According to research reports, lycopene may reduce the production of advanced glycation end products (AGE) and receptors for advanced glycation end products (RAGE), which help protect vessels [120,144,145]. In the group of tested men, it was also shown that a high level of lycopene is associated with, among other things, inhibiting the atherosclerotic process in the carotid arteries by reducing the thickness of the intima-media complex (IMT). An inverse relationship was demonstrated between the concentration of lycopene and the level of one of the markers of the atherosclerotic process, C-reactive protein (CRP). Reducing its level has a direct impact on the rate of formation of atherosclerotic plaque [146].

A positive effect on the lipid profile is achieved by consuming a minimum of 25 mg of lycopene per day [147,148]. Supplementation with 60 mg of lycopene daily for 3 months leads to a 14% reduction in LDL levels [149]. Lycopene and tomato products have been found in clinical trials to lower total cholesterol and low-density lipoprotein cholesterol (LDL-C). In healthy postmenopausal women, lycopene supplementation can reduce total and LDL cholesterol levels [124,150,151,152].

Moreover, lycopene helps maintain normal blood pressure values, and its effect is greater in the group of subjects with systolic blood pressure values above 140 mm Hg [148]. Consuming lycopene above 12 mg/day reduces systolic blood pressure by an average of 4.95 mm Hg [153]. In people with hypertension, short-term therapy with antioxidant-rich tomato extract (250 mg/day for 8 weeks) can lower blood pressure. The research also showed that after 8 weeks of lycopene supplementation at a dose of 15 mg daily, there was a significant reduction in blood pressure. Lycopene reduces oxidative stress and indirectly increases the production of nitric oxide (NO) in the endothelium, acting as an antioxidant and lowering blood pressure. After 6 weeks of supplementation with tomato extract, in the study group, in people suffering from moderate hypertension and already using angiotensin-converting enzyme inhibitors (ACE inhibitors) or calcium channel blockers, a significant reduction in both systolic and diastolic blood pressure was found, which indicates the important role of lycopene in the treatment of hypertension [120]. Lycopene supplementation (above 12 mg/day) reduces systolic blood pressure in patients with prehypertension and hypertension but does not affect diastolic blood pressure [120,154,155]. Lycopene can inhibit the conversion of angiotensin I to angiotensin II by inhibiting inhibitors of the angiotensin-converting enzyme ACE [120]. Due to its antioxidant and anti-inflammatory properties, lycopene supplementation prevented changes in hemodynamic parameters, changes in biochemical and inflammatory markers, and apoptotic changes and reduced the extent of myocardial infarction. It was also shown that in people with the highest level of lycopene in adipose tissue (0.62 g/g of collected adipose tissue), the risk of heart attack was 48% lower compared to the group with the lowest level (0.11 g/g) [119,156].

According to studies, a high concentration of lycopene in serum (>0.22 mol/L) reduces the risk of ischemic stroke by 59% compared to the group with lower concentrations (0.030 mol/L). Also, the risk of hemorrhagic stroke is reduced by 55% in the group with high serum lycopene concentrations compared to the group with the lowest concentrations [157]. It has also been shown that regular consumption of lycopene (19 mg/day) reduces the risk of stroke by an average of 19.3% [158].

Further studies involving longer durations of lycopene supplementation are needed to determine the specific correct dose, as many studies showed different benefits at different doses, but there are no clear criteria to determine the exact dose for a specific disease, so only dietary supplements are currently available on the market, and there are no medications.

- Other tomato fractions

Apart from lycopene, other tomato fractions also have interesting applications. In a recent publication [159], the authors describe an innovative method for obtaining oligomers of cutin—a natural biopolymer found in tomato peels—directly from TP. The studies demonstrated that the obtained oligomers exhibit strong antimicrobial properties, opening up prospects for their use in the pharmaceutical industry as natural antibacterial agents. The isolation method is a single-factor, simplified, and effective approach, promoting scalability and the ecological disposal of tomato waste—a critical factor for the sustainable development of the pharmaceutical industry. In short, tomato extracts derived from cutin can act as bioactive additives in pharmaceuticals, contributing to the creation of natural, biodegradable, and safe antimicrobial agents.

## 8. The Use of Tomato Oil in the Cosmetic Industry

According to the International Nomenclature of Cosmetic Ingredients (INCI) and in the official documents of the European Commission (cosmetic ingredients database: CosIng) [160], tomato raws (extracted directly from the plant or its cells) appear under eighteen names: four of them refer to lipophilic fractions obtained from fruit or seeds (*Solanum lycopersicum* Fruit Lipids, *Solanum lycopersicum* Fruit Oil, *Solanum lycopersicum* Seed Oil, *Solanum lycopersicum* Skin Wax), while the others include various extracts (see Table 6 for details).

According to the official User Guide [161], CosIng is “the online consultation tool of the European Commission describing cosmetic ingredients contained in:

- Cosmetics Regulation (EC) No 1223/2009 of the European Parliament and of the Council;
- the Inventory of Cosmetic Ingredients, as amended by Decision 2006/257/EC establishing a common nomenclature of ingredients employed for labeling cosmetic products throughout the EU; and
- opinions on cosmetic ingredients of the Scientific Committee on Consumer Safety.”

Table 6 was prepared based on the CosIng ingredient index. It contains a list of tomato cosmetic raw materials, each accompanied by a brief description. It is worth noting that tomato fruit and tomato cells are also used as cosmetic ingredients [159]. Table 6 does not include raw materials containing post-fermentation filtrates in which one of the ingredients was tomato fruit. In the USA, cosmetic ingredients are laid down in Title 21 of the Code of Federal Regulations (CFR) reserved for rules of the Food and Drug Administration [162]. In 2017, the Cosmetic Ingredient Review (CIR) Expert Panel assessed the safety of *Solanum lycopersicum* Seed Oil and *Solanum lycopersicum* Fruit Oil inter alia [163].

The cosmetic products on the market containing tomato fruit/seed oil were analyzed via an Internet search. The search phrase “cosmetics containing Tomato Seed Oil” yielded 65,800,000 results in Google, while the phrase “cosmetics containing Tomato Fruit Oil” yielded only 20,700 results. The search for cosmetics with tomato skin wax brought the biggest surprise: only one result. Among the results were pages referring to cosmetic raw materials, retail sales of the oil, and online stores selling cosmetics with it. Nevertheless, within the initial search results page, there were listings for cosmetics incorporating tomato oil, including one specifically designed for the US market. The Environmental Working Group (EWG), an independent nonprofit organization based in the US and funded through grants from charitable foundations and individual contributions, collected data about 4 cosmetics containing the *Solanum lycopersicum* (Tomato) Fruit Lipids. In contrast, no cosmetics were found with the remaining three lipophilic raw materials. According to the information given by INCI Decoder (science-based ingredient verifying tool), there are 145 cosmetics with *Solanum lycopersicum* Seed Oil (Table 7) but only 4 cosmetics with *Solanum lycopersicum* Fruit Lipids, 1 with *Solanum lycopersicum* Fruit Oil and none with *Solanum lycopersicum* Skin Wax. When analyzing this data, it becomes apparent that skincare products for various purposes are the majority. In second place are diverse types of cleansing products. However, most of them include cleansing oils.

Tomato seed oil is a source of unsaturated fatty acids [57,58]. The properties and uses of the oil are similar to those of tomato pulp—it contains large amounts of carotenoids [62] and fat-soluble vitamins [3,78,90]. There are also amounts of phytosterols and flavonoids [3,70,79,88]. Thanks to these ingredients, tomato seed oil has anti-wrinkle and indirectly moisturizing properties. Although lycopene, according to its absorption characteristics, does not have sun-screening properties, it acts as a scavenger for lipid radicals, mitigates lipid peroxidation, and shields against erythema induced by UV radiation on the skin. Its presence can mitigate the harmful impact of UV light on the skin, enhancing defense against both immediate (sunburn) and long-term consequences of sun exposure (such as cancer) [84,85,164]. Producers of cosmetic raw materials rarely declare the origin of their raw materials. Only in the case of certification such practices are used. In the case of raw materials containing tomato extracts, one company was found that declared the use of TP in the production of its raw materials [165].

## 9. Conclusions

The accumulation, management, and disposal of TP raise significant challenges for the tomato processing industry. Consequently, exploring the potential valorization of TP into high-value products offers not only an economic opportunity for the agri-food sector but also a sustainable approach to addressing waste management issues. This review provides a comprehensive synthesis of current knowledge regarding the compositional characteristics of TP, and it examines the utilization of tomato-derived oil and lycopene in the pharmaceutical and cosmetic industries.

The high lipid content of tomato seeds supports the feasibility of oil recovery through mechanical pressing or other environmentally sustainable extraction techniques, which eliminate the need for solvents and preserve a greater proportion of polar bioactive compounds, such as flavonoids and phenolic acids. The unique compositional profile of tomato seed oil, in combination with the potential enhancement of its functional properties through the incorporation of oleoresins derived from pomace or peel fractions, renders these products suitable for applications in the food, nutraceutical, and cosmetic industries. However, large-scale implementation remains limited, primarily due to a lack of integration across different valorization strategies and the absence of a systematic framework to consolidate these diverse approaches.

This publication places particular emphasis on the extraction of raw materials (e.g., tomato pomace oil, lycopene extract) from TP for use in the cosmetics and pharmaceutical industries while also acknowledging the broader context of TP valorization. Potential avenues for future research and practical implementation strategies are identified. The tomato processing industry represents a critical socio-economic sector within the European Union, generating substantial volumes of waste, including spoiled and unripe tomatoes, stems, seeds, peels, and pomace. Current research efforts focus on the effective management of these byproducts, particularly through the extraction of bioactive compounds such as β-carotene, lycopene, and phenolic compounds, as well as the utilization of anaerobic fermentation for biogas production. Despite extensive studies on the extraction of these compounds, methodological discrepancies and variations in experimental conditions have led to challenges in data comparability. Standardized analytical protocols and normalization procedures are urgently needed to facilitate comparative analyses, converting results into initial biomass mass and extract yield.

Biorefinery-based approaches, designed to maximize resource efficiency while minimizing environmental impact, are increasingly being explored. The integration of multiple processing strategies, including the extraction of bioactive compounds, anaerobic digestion, and composting, has demonstrated promising results. Particular attention is directed toward co-fermentation strategies that incorporate various agricultural residues, thereby enhancing process efficiency and resource utilization in industrial applications.

Techno-economic analyses indicate that recovering carotenoids from tomato waste is potentially profitable; however, further research is needed to evaluate and compare different valorization strategies within the biorefinery industry. Additionally, comprehensive safety assessments, including microbiological evaluations and toxicological screenings, should be conducted to ensure product safety and compliance with relevant regulations. Furthermore, life cycle assessments (LCAs) of tomato waste management are currently lacking, yet they are essential for identifying the most effective processing methods and optimizing resource allocation.

In conclusion, advancing the sustainable management of tomato waste necessitates further comparative studies, in-depth economic evaluations, and extensive life cycle assessments. Such efforts will enhance cost-effectiveness in the tomato processing industry while simultaneously supporting the principles of the circular economy and sustainable resource utilization.

## Figures and Tables

**Table 5:** Lycopene content in dietary supplements.

FORM of the Supplement	Amount of Lycopene
Capsule (1 caps.)	0.33 mg
	0.5 mg
	0.6 mg
	1 mg
	1.25 mg
	2 mg
	2.5 mg
	3.25 mg
	5 mg
	7.5 mg
	10 mg
	15 mg
Pill (1 pill)	0.1 mg
	0.25 mg
	0.5 mg
	1 mg
	15 mg
	25 mg
	30 mg
Chewable tablet (1 chewable tablet)	0.25 mg
Liquid (100 mL)	8 mg
	12 mg

**Table 6:** List of official tomato raw INCI names.

INCI Name	Description According to CosIng
Hydrolyzed Tomato Skin	Hydrolyzed Tomato Skin is the hydrolysate of the skins of *Solanum lycopersicum* derived by acid, enzyme or other method of hydrolysis
*Solanum lycopersicum* Callus Culture Extract	*Solanum lycopersicum* Callus Culture Extract is the extract of a culture of the callus cells of the Tomato, *Solanum lycopersicum* L., Solanaceae
*Solanum lycopersicum* Calyx Extract	*Solanum lycopersicum* Calyx Extract is the extract of the calyxes of *Solanum lycopersicum*
*Solanum lycopersicum* Flower Extract	*Solanum lycopersicum* Flower Extract is the extract of the flowers of *Solanum lycopersicum*.
*Solanum lycopersicum* Fruit	*Solanum lycopersicum* Fruit is the dried fruit of the Tomato, *Solanum lycopersicum* L., Solanaceae
*Solanum lycopersicum* Fruit Extract	*Solanum lycopersicum* Fruit Extract is an extract obtained from the fruit of the Tomato, *Solanum lycopersicum* L., Solanaceae
*Solanum lycopersicum* Fruit Juice	*Solanum lycopersicum* Fruit Juice is the juice expressed from the fruit of the Tomato, *Solanum lycopersicum* L., Solanaceae
*Solanum lycopersicum* Fruit Lipids	*Solanum lycopersicum* Fruit Lipids are the lipids extracted from the fruit of the Tomato, *Solanum lycopersicum* L., Solanaceae
*Solanum lycopersicum* Fruit Oil	*Solanum lycopersicum* Fruit Oil is the oil extrated from the fruit of the Tomato, *Solanum lycopersicum* L., Solanaceae
*Solanum lycopersicum* Fruit Water	*Solanum lycopersicum* Fruit Water is an aqueous solution of the steam distillate obtained from the fruit of the Tomato, *Solanum lycopersicum* L., Solanaceae
*Solanum lycopersicum* Fruit/Leaf/Stem Extract	*Solanum lycopersicum* Fruit/Leaf/Stem Extract is an extract of the leaves, stems and fruit of the Tomato, *Solanum lycopersicum* L., Solanaceae
*Solanum lycopersicum* Leaf Cell Culture Extract	*Solanum lycopersicum* (Tomato) Leaf Cell Culture Extract is the extract of a culture of the leaf cells of Solanum lycopersicum, Solanaceae
*Solanum lycopersicum* Leaf Cell Extract	*Solanum lycopersicum* Leaf Cell Extract is the extract of the leaf cells of *Solanum lycopersicum* grown in culture, Solanaceae.
*Solanum lycopersicum* Leaf Extract	*Solanum lycopersicum* (Tomato) Leaf Extract is the extract of the leaves of *Solanum lycopersicum*.
*Solanum lycopersicum* Meristem Cell	*Solanum lycopersicum* Meristem Cell are the meristem cells isolated from the Tomato, *Solanum lycopersicum* L., Solanaceae
*Solanum lycopersicum* Seed Extract	*Solanum lycopersicum* Seed Extract is the extract of the seeds of the tomato *Solanum lycopersicum*
*Solanum lycopersicum* Seed Oil	*Solanum lycopersicum* Seed Oil is the fixed oil obtained from the seeds of the Tomato, *Solanum lycopersicum* L., Solanaceae
*Solanum lycopersicum* Skin Wax	*Solanum lycopersicum* Skin Wax is the wax derived from the skin of the tomato, *Solanum lycopersicum*.

The names of cosmetic ingredients are presented in the INCI format, as officially spelled, without abbreviations or translations [160].

**Table 7:** Summary of products containing *Solanum lycopersicum* Seed Oil based on INCI Decoder data.

Product Type	Number of Products
facial moisturizer/treatment	42
facial cleanser	19
sunscreen	18
serums/essences	16
facial mask	13
moisturizer	8
hair conditioner/serum	8
eye cream/treatment	5
toners	4
body wash/cleanser	3
hand cream	3
lip balm	3
pad	2
scrub	1

INCI name used verbatim: *Solanum lycopersicum* Seed Oil.

## Data Availability

Not applicable.

## Abbreviations

The following abbreviations are used in this manuscript:

TP	Tomato pomace
FAO	Food and Agriculture Organization
SDG	Sustainable Development Goal
RSM	Response surface methodology
ROS	Reactive oxygen species
VCAM-1	Vascular cell adhesion protein-1
sVCAM	Circulating Vascular Cell Adhesion Molecule
sICAM	Soluble intracellular adhesion molecules
SOD	Superoxide dismutase
INCI	International Nomenclature of Cosmetic Ingredients
CFR	Code of Federal Regulations
CIR	Cosmetic Ingredient Review
EWG	Environmental Working Group
